# Recent Advances in Clinical Glycoproteomics of Immunoglobulins (Igs)*

**DOI:** 10.1074/mcp.O116.058503

**Published:** 2016-03-23

**Authors:** Rosina Plomp, Albert Bondt, Noortje de Haan, Yoann Rombouts, Manfred Wuhrer

**Affiliations:** From the ‡Leiden University Medical Center, Center for Proteomics and Metabolomics, Leiden, The Netherlands;; §Leiden University Medical Center, Department of Rheumatology, Leiden, The Netherlands;; ¶Institut de Pharmacologie et de Biologie Structurale, Université de Toulouse, CNRS, UPS, France

## Abstract

Antibody glycosylation analysis has seen methodological progress resulting in new findings with regard to antibody glycan structure and function in recent years. For example, antigen-specific IgG glycosylation analysis is now applicable for clinical samples because of the increased sensitivity of measurements, and this has led to new insights in the relationship between IgG glycosylation and various diseases. Furthermore, many new methods have been developed for the purification and analysis of IgG Fc glycopeptides, notably multiple reaction monitoring for high-throughput quantitative glycosylation analysis. In addition, new protocols for IgG Fab glycosylation analysis were established revealing autoimmune disease-associated changes. Functional analysis has shown that glycosylation of IgA and IgE is involved in transport across the intestinal epithelium and receptor binding, respectively.

Glycosylation of immunoglobulins (Igs) plays a key role in the regulation of immune reactions: glycans located at various sites modulate a diversity of immunoglobulin properties including protein conformation and stability, serum half-life, as well as binding affinities to antigens, receptors and glycan-binding proteins (GBP)^1^ (1–3).

The five classes of human antibodies—IgG, IgA, IgM, IgE, and IgD—each contain one to six sites for *N*-linked glycosylation within the conserved sequence of each heavy chain (4). IgA1, IgD, and IgG3 also carry *O*-linked glycans on their hinge-region (4, 5). In addition, immunoglobulins can be glycosylated in the variable domain of the Fab (antigen-binding fragment) (6–8). Importantly, glycosylation adds a formidable degree of complexity to protein species, because a range of glycan structures is usually present at each glycosylation site.

Studies on the functional consequences of immunoglobulin glycosylation, especially for IgG, have shown that glycans linked to the Fc (fragment crystallizable) part of the antibody influence the interaction with Fc receptors and GBPs, thereby regulating the pro- or anti-inflammatory immune response (1, 9–12). For example, lack of a fucose on the IgG Fc glycan can enact a 100-fold increase in antibody-dependent cellular cytotoxicity (ADCC) (13, 14). Fc-linked glycans may also influence the endocytosis, transcytosis and half-life of some classes of immunoglobulin, such as IgA (15, 16). Next to Fc-linked glycosylation, glycans attached to the Fab region also influence Ig properties and inflammation, especially by modulating antigen recognition and antibody aggregation, as well as through the binding to GBP (7, 17). Importantly, antibody glycosylation has been shown to reflect the physiological and pathological condition of an organism (18–20).

Because of the impact on the immunological response and thus the efficacy of therapeutic antibody treatment, it is crucial to monitor and in some cases alter the glycosylation profile in order to optimize antibody effector functions (9, 13). Glycosylation of antibodies can vary widely depending on the expression system and cell culture conditions during production (13). Because nonhuman glycan structures can trigger immunogenic responses, therapeutic antibodies are currently produced exclusively in mammalian cell cultures. Because of improvements in glyco-engineering, it is expected that non-mammalian expression systems will soon be applicable as well (13, 21). Robust and high-throughput methods are needed to monitor the glycosylation of therapeutic antibodies. Additionally, glycosylation analysis should be site-specific because the function of a glycan can depend on its location, as illustrated by the different influence of glycans located at the Fc and at the Fab part of IgG (12, 17).

Glycosylation profiling of antibodies is usually done using one of the following approaches: (1) by releasing glycans from the protein, which is easily done for *N*-glycans by digestion with PNGase F, whereas *O*-glycans can be released chemically through hydrazinolysis or beta-elimination; (2) by using a proteolytic enzyme to digest the glycoprotein, resulting in glycopeptides; or (3) by analyzing the intact glycoprotein or portions thereof (*e.g.* Ig heavy and light chains) (22–24). Recent years have seen major methodological advances in all three approaches as detailed in this review. In addition, selected examples are given of antibody glycosylation studies in both biotechnological and biomedical research.

In the field of immunoglobulin (glyco)proteomics, several nomenclatures for the glycosylation sites are used (Table I). The one most commonly used refers to the Asn positions as determined in the old days based on Edman sequencing of both variable and heavy chains (*e.g.* (4)). Alternatively, the homology-based nomenclature by the international ImMunoGeneTics information system (IMGT) is available for immunoglobulins, which has the advantage of a more intuitive comparison between the different immunoglobulins (*e.g.* site homology between CH2 84.4 on IgG and IgD, as well as similarity with CH3 84.4 on IgE and IgM) (25). In this review we will use the UniProt based site annotation, because this is more easily integrated with proteomic databases (26).

**Table I:** Several different immunoglobulin protein sequence nomenclatures are used in literature. The nomenclature most frequently used in literature is based on archaic sequencing data of both immunoglobulin variable and constant domains, whereas the UniProt numbering is based on the conserved sequences, and the IMGT nomenclature is based on homology between the immunoglobulins

	Conventional literature^a^	UniProt^b^	IMGT^c^
IgG1	297	180	CH2-84.4
IgG2	297	176	CH2-84.4
IgG3	297	227	CH2-84.4
IgG3	392	322	CH3-79
IgG4	297	177	CH2-84.4
IgA1	263	144	CH2-20
IgA1	459	340	CHS-7
IgA2	166	47	CH1-45.2
IgA2	211	92	CH1-114
IgA2	263	131	CH2-20
IgA2	337	205	CH2-120
IgA2	459	327	CHS-7
IgM	171	46	CH1-45
IgM	332	209	CH2-120
IgM	395	272	CH3-81
IgM	402	279	CH3-84.4
IgM	563	439	CHS-7
IgE	140/145^d^	21	CH1-15.2
IgE	168/173^d^	49	CH1-45.2
IgE	218/219^d^	99	CH1-118
IgE	265	146	CH2-38
IgE	371	252	CH3-38
IgE	394	275	CH3-84.4
IgD	354	225	CH2-84.4
IgD	445	316	CH3-45.4
IgD	496	367	CH3-116

^a^ As used in e.g. (4).

^b^ (26).

^c^ (25).

^d^ alternative nomenclature used in (121).

Analysis of the antibodies themselves is complicated by the variable domain that dictates the specificity of the antigen-binding site. Protein sequencing of monoclonal antibodies or affinity-purified antibodies is done using high resolution liquid chromatography tandem mass spectrometry methods, coupled to DNA sequence information generated by next-generation sequencing of the B-cell antibody repertoire (27, 28). Post-translational modifications, such as glycosylation, further complicate antibody analysis and require specific analysis strategies, as will be detailed in this review.

## 

### 

#### IgG-Fc Glycosylation at Asn180/176/227/177 ('Asn297')

The majority of IgG glycosylation analysis has been focused on the Fc glycan because of both the known influence of this glycan on IgG effector functions and the established high-throughput methods that are available to selectively monitor this glycosylation site (12, 23, 24). In human IgG, the conserved *N*-glycosylation site is located at Asn180 (IgG1; UniProt P01857), Asn176 (IgG2; P01859), Asn227 (IgG3; P01860), or Asn177 (IgG4; P01861), alternatively referred to as position CH2–84.4 (25) or Asn297 (*e.g.* in (4); Table I). In all IgG subclasses, the Fc-glycosylation site has been shown to harbor complex type diantennary *N*-glycans that carry between zero and two galactoses, with the majority carrying a core fucose, and a minority having a bisecting *N*-acetylglucosamine (GlcNAc) and one or two sialic acids (29). The glycan at this site has been shown to influence the inflammatory capacity of IgG through modulation of the binding to Fc-gamma receptors (FcγRs) and C-type lectins: in general, the absence of a core fucose and/or absence of galactoses and sialic acids appear to convey pro-inflammatory properties, whereas the presence of terminal sialic acids triggers an anti-inflammatory response (1, 12, 30).

Changes in Fc glycosylation, *i.e.* a decrease in galactosylation and sialylation that contributes to a more inflammatory antibody profile, have been observed in various autoimmune disorders, most recently inflammatory bowel disease (IBD), systemic lupus erythematosus (SLE), multiple sclerosis (MS) and chronic inflammatory demyelinating polyneuropathy (CIDP) (31–34). In addition to autoimmune disorders, IgG glycosylation changes can also occur in infectious diseases, as was shown by recent studies on HIV infection, chronic hepatitis B and the parasitic disease visceral leishmaniasis (35–37). Furthermore, new reports reaffirm the potential role for IgG glycosylation as a biomarker for cancer progression (38, 39). Finally, congenital defects in glycosylation or carbohydrate metabolism also alter IgG glycosylation, as shown recently for Man1B1 deficiency and galactosemia (40, 41).

#### Analysis of Released Glycans

The gold standard for studying IgG glycosylation relies on enzymatic *N*-glycan release, subsequent fluorescent labeling by reductive amination and analysis of the labeled glycans by high-performance liquid chromatography (HPLC) using hydrophilic interaction liquid chromatography (HILIC) with fluorescence detection (42). First, this approach has been further developed by implementing HILIC stationary phases for ultra-performance liquid chromatography (UPLC) instrumentation, thereby improving both throughput and resolution (43). Second, sample preparation has been simplified through the use of 96-well filter plates to increase the throughput of glycan purification (44), as well as by introducing fluorescent tags to label the glycosylamine species released by PNGase F, instead of targeting the aldehyde species that arise from acid-catalyzed hydrolysis of the glycosylamine (45). Of note, the increased throughput capacity allowed for analyses of large sample sets, which could for example show the potential of the IgG *N*-glycans as a marker of chronological and biological age (46). Third, sample preparation has been robotized, resulting in a highly automated, higher-throughput workflow and leading to more robust results (45). However, this method has a disadvantage: because the glycans are released from the IgG, it is impossible to distinguish between Fab and Fc glycans as well as between glycans originating from different IgG subclasses.

Next to HILIC UPLC of fluorescently labeled glycans, various methods for repetitive IgG glycosylation analysis (“profiling”) have reached maturity as evidenced by the high consistency of the results obtained in extensive method comparison studies (22–24). Remarkably, various mass spectrometric methods showed very good performance with respect to resolution, sensitivity, and robustness, which opened the way to their broad application in both biotechnological (23, 24) and biomedical applications (22).

#### Analysis of Glycopeptides

A bottom-up proteomics approach, with trypsin digestion followed by liquid chromatography (LC) coupled to mass spectrometric analysis, is most commonly applied for site-specific analysis of IgG Fc glycosylation (23). Tryptic digestion results in distinct glycopeptides that allow discrimination of the different IgG subclasses—with the peptide moieties EEQYNSTYR for IgG1, EEQFNSTFR for IgG2, and EEQFNSTYR for IgG4. The peptide sequence of the IgG3 glycopeptide shows allotype variation in the amino acid at the position *N*-terminal of the Asn227, causing a mass that is identical to either the IgG2 peptide (EEQFNSTFR; predominant allotype in Caucasian populations) or the IgG4 sequence (EEQYNSTFR; predominant allotype in Asian and African populations) (47). Although trypsin digestion forms the gold standard for Fc IgG glycopeptide analysis, we have recently found that incomplete denaturation and digestion of IgG might lead to biases in glycoprofiling (48).

To prevent ion suppression of the glycopeptides by unglycosylated peptides during mass spectrometric analysis, a glycopeptide separation or enrichment step is often applied. This separation is usually performed using either reverse phase LC with C18 or graphitized carbon as a stationary phase, or by HILIC (2). Although a variety of stationary phases exist for HILIC, amide is most frequently used, although polysaccharide-based stationary phases may show similar performance. Notably, the affinity of current HILIC materials is often dependent on glycan structure, which can lead to a bias in enrichment (2). The lack of a gold standard method has led to the development of various new HILIC materials for glycopeptide enrichment of tryptic IgG (glyco)peptides, with stationary phases consisting of various polysaccharides (chitosan, dextran, cyclodextrin, maltose) coupled to magnetic particles, silica particles or metallo-organic frameworks (49–52), or functionalized amide polymers embedded in a monolith capillary (53). Furthermore, electrostatic repulsion HILIC (also known as ERLIC) has been successfully employed to enrich IgG glycopeptides, although a thorough analysis of the potential skewing of IgG glycoforms in ERLIC enrichment is still lacking (54). Zwitterionic (ZIC) HILIC has also gained popularity: this technique makes use of highly hydrophilic materials carrying both positive and negative charges and shows a very good performance in glycopeptide enrichment (55). Novel ZIC HILIC materials, consisting of zwitterionic polymers coupled to silica particles or magnetic nanoparticles, have been developed and feature high sensitivity in post-enrichment MALDI-MS measurements (56–58).

Multiple reaction monitoring (MRM) is well-suited for high-throughput quantitative analysis of complex samples and has only recently been applied to glycopeptide Ig analysis (59–61). Hong *et al.* developed a method that simultaneously performs glycoprofiling of IgG and absolute quantitation of IgG and its separate subclasses, which can help determine if a relative change in glycosylation is because of changes in post-translational modification or changes in the level of protein production (61). For the quantification of glycopeptides, MRM was set to specifically detect oxonium ions, fragment ions that originate during the fragmentation of glycopeptides. Similar methods are being developed for IgA and IgM (59). A comparable protocol describing MRM detection of IgG glycoforms was separately developed by Yuan *et al.*, with the added feature of a prior separation of IgG3 so that glycosylation could be observed separately for each of the four IgG subclasses (62).

Capillary electrophoresis (CE) coupled to MS has likewise been applied for tryptic IgG analysis and shows a vastly increased sensitivity compared with the LC-MS approach (63). The sensitivity gain may be largely ascribed to the very low flow rates achieved in CE-MS.

Compared with LC-ESI-MS or CE-MS, MALDI-MS offers higher sample throughput as well as lower data complexity. However, detection of glycopeptides is complicated by the loss of sialic acids through in-source decay. Of note, this can be prevented by neutralizing the charge on the sialic acid by (dimethyl-)amidation (29, 64) or ethyl esterification (65). The derivatization methods target carboxyl groups on the peptide moiety as well as on the glycan, which can provide useful structural information from a combination of positive- and negative-ion MS/MS analyses (64). An additional advantage of these methods is the introduced mass difference between α2,3- and α2,6-linked sialic acids, caused by the sialic acid linkage-specificity of the reactions. Although the ethyl esterification method is highly specific for sialic acid linkages on the level of released glycans (66), the modification of the peptide moiety of glycopeptides was found to be not completely specific, resulting in unwanted byproducts. This issue has been addressed and overcome by a recently published method using dimethylamidation, which provides sialic acid linkage information on a stably modified glycopeptide (Fig. 1) (29). Currently, the dimethylamidation of sialylated glycopeptides is optimized for IgG Fc-glycopeptides and would need further optimization when used for different glycoproteins, such as other immunoglobulins. MALDI-TOF-MS/MS of sialylated glycopeptides, using laser induced fragmentation, highly benefits from the derivatization, as the loss of the sialic acid is no longer the dominant fragment. In addition to analysis with MALDI-TOF-MS(/MS), the derivatization method has been shown to be applicable for the analysis of IgG Fc-glycopeptides, using LC-MS(/MS), enabling differentiation between differently linked sialic acids, without having a major influence on the fragmentation of the analytes (data not published). Pyrene derivatization is an alternative method for glycopeptide analysis by MALDI-MS and also allows discrimination between α2,3- and α2,6-linked sialic acids, although its application on immunoglobulins has not been established (67, 68).

**Fig. 1.**
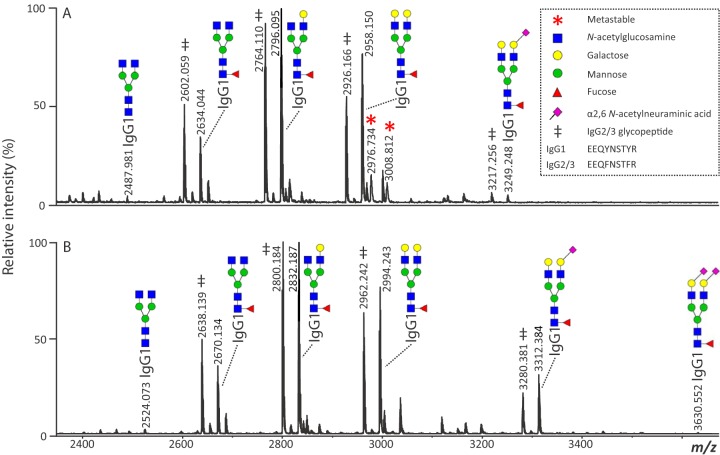
**MALDI-TOF-MS spectra of human plasma IgG Fc-glycopeptides (*A*) without derivatization, and (*B*) after dimethylamidation.** Derivatization results in the stabilization of the sialylated glycopeptides, improving their detection and preventing the formation of metastable signals. With the use of dimethylamidation, no unspecific side reactivity on the peptides was observed and the linkage of the sialic acids could be determined (29).

Furthermore, stable isotope labeling of glycopeptides has recently been achieved: succinic anhydride was used to introduce a mass increment of 8 Da to tryptic IgG1 glycopeptides (69). This method can be used to perform absolute quantification of IgG glycopeptides, or can be used to reduce bias during sample analysis by parallel analysis of two samples. Isobaric labeling using tandem mass tags (TMTs), which lead to different masses upon fragmentation, has also been used for the analysis of pepsin-generated IgG glycopeptides (70).

#### Analysis of Intact Glycoprotein or Glycoprotein Fragments

Alternatively, a middle-down or middle-up proteomics approach can be applied for assessing IgG Fc glycosylation. The Fc portion can be cleaved from the Fab portion in whole IgG, or from fused proteins in Fc-fusion proteins, by limited proteolytic digestion with the protease IdeS (FabRICATOR®), followed by either mass spectrometric analysis of the protein fragment or purification of the Fc portion and release of the glycans using PNGase F (6, 71–73). The latter method has recently been applied to a clinical sample set, as is discussed later in this review (6).

Finally, technical advances in the recent years have allowed for top-down mass spectrometric analysis of monoclonal antibodies, allowing the integrated analysis of post-translational modifications (23, 73–76). An extensive review has been recently published by Zhang *et al.*, describing several applications of these techniques, and a comparison between “normal” and native MS, furthermore including ion mobility (77). More recently, ultrahigh resolution machinery (*e.g.* Fourier transform ion cyclotron resonance (FTICR)) allowed for the detection of intact monoclonal antibodies with isotopic resolution, showing several glycoforms (75). Additional top-down MS/MS information was obtained in conjunction with online electrochemical reduction of the antibody (75). Furthermore, isobaric labeling has been applied to intact antibody-drug conjugates (70). Native MS is often applied to analyze intact mAbs (76). The advantage of this approach is the limitation of charge states because of the native 3D protein configuration, causing increased signal-to-noise for the few charge states that do occur. The downside of native MS is a lack of information regarding the glycosylation site(s) or the precise structure of the glycan(s) (76, 77).

#### Fab Glycosylation

The structural features of IgG Fab glycosylation and its emerging importance in immunity have been recently reviewed (17). It has been estimated that ∼15–25% of serum IgG of healthy individuals contain *N*-glycosylation sites and carry *N*-glycans (Fab-glycosylation) in their variable domains, in addition to the almost fully occupied IgG Fc *N*-glycosylation site (78, 79). Of note, the percentage of Fab-glycosylation and glycan structures varies during certain pathological and physiological conditions, as shown in RA, lymphoma, and pregnancy (6, 7, 80, 81). Because only a few germline-encoded sequences contain an *N*-glycosylation site, the sites present in the variable domains of immunoglobulins are mainly introduced by somatic hypermutation during the process of affinity maturation (8, 82). Within an affinity-purified population of antigen-specific IgG, identification of Fab glycosylation sites has been achieved by labeling the sites with ^18^O during deglycosylation with PNGase F. This was followed by mass spectrometry-assisted proteomics analysis that revealed the mass shift denoting the site of glycosylation and the peptide sequence surrounding the site (7). The human immune system comprises of an enormous antibody repertoire, recognizing an estimated billion or more different antigens. Antibody specificity is determined by a unique amino acid sequence in the Fab portion, thus making the analysis of Fab *N*-glycopeptides derived from polyclonal antibodies very difficult, if feasible at all.

Therefore, in order to analyze polyclonal IgG Fab glycosylation, the currently used analytical methods consist of the release of *N*-glycans from purified Fab fragments followed by their analysis using capillary electrophoresis with laser-induced fluorescence detection (CE-LIF), (ultra)high performance liquid chromatography and/or mass spectrometry (6, 7, 83). In order to improve the throughput of IgG Fab glycosylation analysis, we recently set up a new sample preparation method. The method relies on IgG affinity capturing in a 96-well filter plate, on-bead proteolytic release of the Fab portions, and collection of Fab (flow-through) and Fc portions (eluate) followed by enzymatic glycan release. Detailed glycan information was obtained by MALDI-TOF-MS after sialic acid stabilization. The method was applied to study the differences between Fab and Fc glycosylation in young women, and the pregnancy associated changes thereof (6). The levels of galactosylation, sialylation, and bisection were significantly higher on the Fab portion compared with the Fc. During pregnancy Fab and Fc glycans showed similar patterns in their changes. Interestingly, the Fab portion was also shown to carry minor amounts of α2,3-linked sialic acids. In general, the *N*-glycans on the Fab are more extended, and some species seem to be Fab-specific. Diantennary fucosylated glycans with two sialic acids are hardly present on the Fc, whereas they are the major species on Fab. Similarly, the presence of a bisecting GlcNAc on glycans with two galactoses appears to be more prominent in Fab *versus* Fc (6). Of note, these data result from the analysis of solely young women, whereas characterization of Fab glycosylation in males as well as different age groups is still lacking.

High-sensitivity analysis of released Fab glycans can also be performed by CGE-LIF (83). However, the glycosylation data obtained via these techniques, especially regarding both the levels of *N*-glycan bisection in IgG Fab portions and various glycosylation features of murine IgG, has shown some discrepancies as compared with results obtained with other analytical methods (6, 84, 85). Additional studies are needed to further unravel Fab glycosylation changes with age, sex, and diseases.

#### Additional N- and O-glycosylation of IgG3

In addition to the well-known Fc *N*-glycosylation site, several allotypes of IgG3 possess a second *N*-linked site in the CH3 domain at Asn322 (UniProt P01860; alternatively referred to as CH3–79 (25) or Asn392; Table I) (86). Only 10% of Asn322 was found to be occupied; the *N*-glycans found at this site were mainly complex type diantennary structures, which differ from those at Asn227 in that the majority is afucosylated and contains a bisecting GlcNAc, and a minority of high mannose type *N*-glycans is also present (86). Because trypsin digestion produces a very large glycopeptide containing this site, the glycan structures may alternatively be examined after digestion with aspecific proteases. Aspecific proteases such as Pronase are particularly useful for the study of glycoproteins because they tend to produce small glycopeptides that are well suited for mass spectrometric analysis (87, 88). Sequential chromatography of resulting digests on a C18 reversed phase column and a porous graphitized carbon column provides the broad retention range necessary to observe all glycopeptides regardless of the heterogeneous retention properties of both the glycan and the peptide moiety. Using collision-induced dissociation with a combination of lower- and enhanced-energy, which produced both glycan and peptide fragmentation, respectively, identification of both the glycan and the peptide moiety in one run could be achieved (86).

Next to *N*-glycosylation, IgG3 may carry up to 3 *O*-glycosylation sites per heavy chain within a triple repeat in the hinge region (5). Proteolytic digestion with trypsin, proteinase K or Pronase followed by LC-MS/MS analysis revealed that ∼10% of each of these sites is occupied, mainly by sialylated core 1 type *O*-glycan structures (5, 86). As both the Fc *N*-glycosylation at Asn322 and the *O*-glycans of IgG3 have been described only recently, their function and clinical relevance remain to be investigated.

#### Antigen-specific IgG

The glycoprofiling of antigen-specific antibodies in clinical samples after vaccination or during disease started less than a decade ago. This was made possible by the numerous improvements in sensitivity and throughput of methods for both antibody purification and glycosylation analysis. Antigen-specific antibodies are generally purified by affinity chromatography using antigens coated on 96-well plates or on chromatography beads/columns. Antigens are usually synthetic peptides or recombinant (glyco)proteins. For instance, the high-throughput purification of anti-citrullinated peptide/protein antibodies (ACPA), *i.e.* autoantibodies specific for rheumatoid arthritis (RA), has been achieved by repeated capturing on 96-well plates coated with a synthetic circular peptide containing citrulline, called CCP2 (cyclic citrullinated peptide 2) (19, 89, 90). Likewise, antibodies directed against multiple HIV and influenza antigens (*e.g.* HIV gp41 and gp120 or influenza hemagglutinin) have been enriched using amino-link antigen resin or antigen-functionalized streptavidin resins packed into cartridges (36, 91, 92). Alternatively, antigen-specific antibodies can be captured by using viral particles, microorganisms or cells. Thus, Vidarsson and coworkers have isolated anti-platelet antibodies (causing neonatal alloimmune thrombocytopenia) and anti-red blood cell antibodies (responsible for hemolytic disease of the fetus and newborn) by incubating serum of pregnant women directly on platelets and red blood cells (93–95). Fc- and/or Fab-linked glycosylation of antigen-specific IgG has been analyzed either at the glycopeptide level mainly using LC-MS, or by releasing glycans using a middle-down/middle-up proteomics approach as described above. Of note, unlike glycopeptide detection, analysis of released glycans from antigen-specific IgG requires another purification step prior to or after antigen-specific capturing in order to separate IgG from other serum glycoproteins or other immunoglobulins (91).

Antigen-specific IgG displays different sialylation, galactosylation, fucosylation, and/or bisection patterns compared with total IgG isolated from the same individuals. Importantly, these structural differences are clearly associated with clinical and functional consequences including disease outcome, disease severity and/or antiviral control responses. For instance, as compared with total IgG, anti-platelet IgGs found in the serum of pregnant women exhibit an exceptionally low level of fucosylation in their Fc-glycans, which enhances the binding affinity for the FcγRIIIa/b and the phagocytosis of platelets, and correlates with increased severity of neonatal alloimmune thrombocytopenia (95). Likewise, HIV-specific IgG antibodies isolated from HIV-positive subjects present a higher frequency of afucosylated, agalactosylated, and asialylated *N*-glycans compared with total IgG. Importantly, this glycan difference, especially the greater percentage of agalactosylated glycoforms, is far more pronounced in HIV elite controllers than in (un)treated chronic progressors and is associated with an enhanced capacity to bind to FcγRIIIa, probably explaining the more potent antibody-dependent cellular viral inhibition activity that characterizes antibody from elite controllers (36). A disruption in the balance between type I (part of the Ig receptor superfamily that includes FcγRI, II and III) and type II (C-type lectin receptors) Fc receptor signaling also very likely occurs in several autoimmune diseases such as rheumatoid arthritis and granulomatosis with polyangiitis (GPA), in which changes in autoantibody-specific glycosylation have been observed. Thus, the Fc-galactosylation, sialylation, and bisection of anti-proteinase 3 (PR3) antibodies IgG1 are reduced compared with total IgG1 in GPA patients (96). Despite an early study reporting a negative correlation between the level of anti-PR3 specific IgG sialylation and disease activity as measured by the Birmingham Vasculitis Activity Score (BVAS), recent evidence demonstrated that the BVAS is strongly associated with the presence of bisecting GlcNAc on anti-PR3 IgG but not with galactosylation/sialylation percentages (96, 97). Interestingly, the level of anti-PR3 IgG galactosylation was associated with pro-inflammatory cytokineconcentrations and time to remission (96). Similarly, in RA patients, ACPA-IgG autoantibodies exhibit a decrease in Fc galactosylation and sialylation levels that occurs a few months before disease presentation, correlates with disease severity, and potentially determines osteoclast differentiation and bone loss during RA (19, 89, 98). Variations in antigen-specific IgG glycosylation have also been observed following vaccination and, more importantly, can predict the efficacy of vaccination (92, 99). Ravetch and coworkers recently showed that the sialylated Fc glycan abundance on anti-hemagglutinin IgG produced by day 7 following influenza virus vaccination predicts the quality of the vaccine response (92). It was proposed that immune complexes formed with Fc-sialylated IgG signal through the type II FcR CD23 on activated B cells and triggers the expression of FcγRIIb, thereby driving the selection of higher affinity B cells and the generation of higher affinity and more protective anti-HA IgG (92).

#### IgA

There are two subclasses of immunoglobulin A (*i.e.* IgA1 and IgA2), and two known allotypes for the IgA2 subclass (*i.e.* A2m(1) and A2m(2)). IgA1 contains a slightly elongated hinge region compared with IgA2. This elongated hinge contains nine potential *O*-glycosylation sites, of which up to six have been reported to be occupied (100). In addition, IgA1 harbors two *N*-glycosylation sites at Asn144 and Asn340 (UniProt P01876; alternatively referred to as CH2–20 and CHS-7, respectively (25), or Asn263 and Asn459 (*e.g.* in (4)); Table I), whereas IgA2 harbors four sites at Asn47, Asn131, Asn205, and Asn327 (UniProt P01877; also referred to as CH1–45.2, CH2–20, CH2–120, CHS-7 (25), or Asn166, Asn263, Asn337, and Asn459 (*e.g.* in (4)); Table I). In the A2m(2) allotype of IgA2 an additional consensus sequence is present because of the replacement of a proline by a serine, thus forming a glycosylation site at Asn92 (CH1–114/Asn211; Table I). The analysis of (s)IgA glycosylation is generally performed at the level of released glycans (101) or by lectin ELISA, although a few glycopeptide-based LC-MS/MS methods have been described (100, 102–105).

For the analysis of tryptic *O*-glycopeptides, the use of FT-MS/MS coupled online to an RP-LC system has been described (100). Electron transfer dissociation, which preferentially fragments the peptide backbone and not the glycan, was used to determine the location of each glycosylation site. By applying a few additional separation steps using less advanced laboratory techniques, others have achieved similar results by MALDI-TOF/TOF-MS (106).

The IgA *N*-glycosylation is less frequently studied, although it may have important functional consequences as demonstrated by the influence of *N*-glycan sialylation on the transportation of secretory IgA across an *in vitro* model of follicle-associated epithelium via binding to Dectin-1 and Siglec-5 (16).

Nowadays, the use of IgA instead of IgG monoclonal antibodies for biopharmaceutical purposes is being explored, with a focus on anti-HIV drugs (107–109). Therefore, several site-specific glycosylation analysis methods have recently been developed. An LC-ESI-MS/MS method primarily developed for the analysis of HIV gp140 has been adapted and applied to secretory IgA1 produced in plants as well as to human IgA (109, 110). In brief, IgA was digested by sequential application of trypsin and GluC after reduction and alkylation. Next, glycopeptide analysis was performed by first identifying the elution position of deglycosylated peptide moieties. Glycopeptides are known to elute a short time ahead of the deglycosylated variant, with some spread because of the various glycans attached. The addition of a buffered formic acid solution to the flow ascertained very close or even identical elution times for glycosylated peptides bearing sialylated structures. A targeted search for the peptide plus potential glycan *m/z* using selected ion chromatograms completed the analysis. Several high mannose type structures were identified using the applied technique. The analysis did not reveal any hinge region *O*-glycosylation, which could be attributed to the production in plants.

The *N*-glycans of IgA are nevertheless still mainly studied at the level of released glycans. For example, the comparison of released glycans from different IgA constructs obtained from various cell lines showed profound differences, especially regarding the level of sialylation, which correlated with the half-life of these antibodies (111).

Of note, the secreted form of IgA consists of a dimer, which forms a complex with the (also glycosylated) joining (J)-chain and the secretory component. The J-chain harbors a single glycosylation site at Asn71 (UniProt P01591; also referred to as Asn48), which appears to be important for IgA dimerization (112). This site bears mainly highly sialylated diantennary *N*-glycans (101, 104). The secretory component is also highly glycosylated, with seven *N*-glycosylation sites at Asn83, Asn90, Asn135, Asn186, Asn421, Asn469, and Asn499 (UniProt P01833; also referred to as Asn65, Asn72, Asn117, Asn168, Asn403, Asn451, and Asn481, *e.g.* in (113); Table I). The protein contains a wide variety of glycan species: di-, tri-, and tetraantennary glycans, bearing all Lewis epitopes (101, 104). It was suggested that these glycans are meant to bind to lectins of bacteria (101).

#### IgM

Human serum IgM mainly circulates as a pentamer of 950 kDa consisting of ten light chains, ten heavy chains and one joining chain (J-chain). Each IgM monomer contains five conserved *N*-glycosylation sites at Asn46, Asn209, Asn272, Asn279, and Asn439 (UniProt P01871; also known as CH1–45, CH2–120, CH3–81, CH3–84.4, CHS-7 (25), or Asn171, Asn332, Asn395, Asn402, and Asn563; Table I) located within the constant region of the heavy chain. In addition, the previously mentioned J-chain contains one *N*-glycosylation site at Asn71 (UniProt P01591). In two recent studies, a site-specific *N*-glycosylation mapping of human serum IgM was achieved by analyzing IgM glycopeptides, generated by trypsin or trypsin/GluC digestion, using either the classical LC-ESI-MS method or a nano-LC-microarray-MALDI-MS platform (114, 115). The latter consists of a nano-LC reverse phase separation of IgM (glyco)peptides, including the J-chain glycopeptide, followed by high frequency droplet-based fractionation of the nano-LC outflow on microarray chips. Each spot on the microarray is then analyzed by MALDI-MS, with or without pre-digestion with PNGase F to remove *N*-glycans. Both studies demonstrated that glycans linked to Asn279 and Asn439 are predominantly oligomannose structures, whereas glycans attached to Asn46, Asn209, and Asn272 mainly consist of complex-type structures (114, 115). The glycosylation site Asn71 of the J-chain also exhibits complex-type *N*-glycans. The main complex-type *N*-glycans found in IgM heavy chains are diantennary species carrying one or two sialic acids, bisecting GlcNAc and/or a core fucose. Minor proportions of oligomannosidic and hybrid-type glycans were also detected on Asn46 (114). Likewise, Asn279 carries 10% of hybrid-type structures, which are also present in very low amount on Asn209. Based on computer modeling of the IgM structure, the clear distinction between glycosylation sites carrying oligomannose structures (on Asn279 and Asn439) or complex-type *N*-glycans (on Asn46, Asn209, Asn272) has been proposed to be the consequence of the low accessibility of glycans on Asn279 and Asn439 for the glycosyltransferase/glycosidases within the Golgi (115). Finally, although the functional aspect of IgM glycosylation on immunity has not been examined yet, the recent possibility of producing human-like glycoengineered heteromultimeric IgM in plants may help to provide new insights in this field (115).

#### IgE

With six oligosaccharides on each heavy chain at Asn21, Asn49, Asn99, Asn146, Asn252, and Asn275 (116) (UniProt P01854; also referred to as CH1–15.2, CH1–45.2, CH1–118, CH2–38, CH3–38, and CH3–84.4 (25), or Asn140, Asn168, Asn218, Asn265, Asn371, and Asn394 *e.g.* in (4); Table I), IgE is the most heavily glycosylated of the immunoglobulins. Characterization of the glycan structures on polyclonal IgE was achieved with a combination of proteolytic enzymes and LC-MS/MS analysis (116). Glycosylation sites Asn21, Asn49, Asn99, Asn146, and Asn252 are occupied by complex type *N*-glycans, which are primarily fully galactosylated diantennary structures, containing a core fucose and one or two sialic acids (116, 117). A high mannose type glycan is present at Asn275, the sixth site, which is homologous to the Fc glycosylation site in IgG. Glycosylation at this site has recently been shown to be essential for the binding of IgE to the high affinity receptor FcεRI and initiation of anaphylaxis (118). Individuals with PGM3-related hyper IgE syndrome or with a hyperimmune condition displayed similar IgE glycosylation compared with healthy individuals (116, 117). Glycosylation analysis of IgE is challenging because of the low concentration in biological fluids: at ∼130–300 ng/ml, the concentration in human serum is roughly 50,000 times lower than that of IgG (119, 120). Because of this limitation, no large-scale glycosylation analysis of IgE in clinical cohorts has been performed as of yet. However, recent advances in LC- and CE-MS sensitivity and robustness may allow for some attempts in the near future.

#### Conclusions and Perspectives

Thanks to the improvement of sample preparation methods and analytical technologies, recent years have seen an increase in sensitivity, accuracy and robustness of IgG glycosylation analysis. These methodological and technological advances are beneficial for biopharmaceutical companies, allowing a better characterization of antibody-based biopharmaceuticals, biosimilars and bio-betters, but are also crucial tools in both basic and clinical research. Thus, this enables, among others, the characterization of glycosylation of antigen-specific IgG, including autoantibodies, alloantibodies and some antipathogen antibodies, which directly impact the immune response and the outcome, progression and/or severity of diseases (19, 36, 89, 91–95, 99). Therefore, methodologies and technologies dedicated to IgG glycosylation analysis have great prospects regarding the early detection and diagnostic of some diseases. Of note, most studies on IgG glycosylation have focused on serum/plasma antibodies, whereas IgG in other biofluids and tissue remain largely unstudied.

In addition to IgG, the substantial recent advances in purifying and analyzing small amounts of samples have helped to analyze the glycosylation of other immunoglobulin subclasses (*i.e.* IgA, IgM, and IgE) in a more precise and comprehensive manner. Today's technological level allows for the simultaneous analysis of multiple immunoglobulin classes in one run (60).

We expect that in the near future, several hiatuses in immunoglobulin related glycomics will be covered. Not only by thorough analysis of the glycosylation of all immunoglobulin classes, but additionally by complementary glycoproteomics analysis of many interacting proteins, such as cell surface derived Fc receptors. This may reveal a regulatory role of both antibody and receptor glycomic variation and the interaction thereof in the regulation of antibody effector functions (14).

## Footnotes

^1^ The abbreviations used are:

GBP: glycan-binding protein
ADCC: antibody-dependent cellular cytotoxicity
CE: capillary electrophoresis
CGE-LIF: capillary gel electrophoresis with laser-induced fluorescence
CH: conserved heavy chain
ERLIC: electrostatic repulsion HILIC
Fab: fragment antigen-binding
Fc: fragment crystallizable
FcγR: Fc-gamma receptor
GlcNAc: *N*-acetylglucosamine
HILIC: hydrophilic interaction liquid chromatography
IgG: immunoglobulin gamma
MRM: multiple reaction monitoring
ZIC HILIC: zwitterionic HILIC.
